# “Not Dead, but Risen” (Poetry)

**Published:** 1878-02

**Authors:** 


### "Not Dead But Risen."

**LINES FROM THE ARABIC, READ BY CHARLES DUDLEY WARNER AT THE MEMORIAL SER[illegible] OF SAMUEL BOWLES.**

He who died at Azim sends
This to comfort all his friends:

Faithful friends! It lies, I know,
Pale and white and cold as snow,
And ye say, "Abdallah's dead!"
Weeping at the feet and head.
I can see your falling tears,
I can hear your sighs and prayers;
Yet I smile and whisper this,—
*I* am not the thing you kiss:
Cease your tears, and let it lie.
It *was* mine, it is not I.

Sweet friends! what the women lave,
For the last sleep of the grave,
Is a hut which I am quitting,
Is a garment no more fitting,
Is a cage from which, at last,
Like a bird my soul has passed.
Love the inmate, not the room—
The wearer, not the garb—the plume
Of the eagle, not the bars
That kept him from those splendid stars!

Loving friends! Be wise and dry
Straightway every weeping eye;
What ye lift upon the bier
Is not worth a single tear.
'Tis an empty sea-shell—one
Out of which the pearl has gone:
The shell is broken—it lies there;
The pearl, the all, the soul is here.
'Tis an earthen jar whose lid
Allah sealed, the while it hid
That treasure of his treasury,
A mind that loved him; let it lie!
Let the shard be earth's once more,
Since the gold is in his store!

Allah glorious! Allah good!
Now thy world is understood;
Now the long, long wonder ends!
Yet ye weep, my erring friends,
While the man whom you call dead,
In unspoken bliss, instead.
Lives and loves you: lost 'tis true,
For the light that shines for you;
But, in the light ye cannot see,
Of undisturbed felicity—
In a perfect paradise,
And a life that never dies.

Farewell friends! But not farewell,
Where I am, ye too shall dwell.
I am gone before your face,
A moment's worth, a little space,
When ye come where I have stept,
Ye will wonder why ye wept;
Ye will know by true love taught,
That here is all and there is naught.
Weep awhile if ye are fain—
Sunshine still must follow rain;
Only not at death—for death,
Now we know, is that first breath
Which our souls draw when we enter
Life, which is of all life centre.

Be ye certain all seems love,
Viewed from Allah's throne above!
Be ye stout of heart, and come
Bravely onward to your home!
La-il Allah! Allah la!
O love divine! O love alway!

He who died at Azim gave
This to those who made his grave.

---

THE DRUGGISTS' HAND BOOK OF PRIVATE FORMULAS. By John Nelson, Livonia Station, N. Y. Mailed post paid on receipt of $3. valuable little book for Druggists, Physicians, and others. It gives [illegible] liable formulas for preparing nearly all the well-known medicines, toil articles, flavoring extracts, syrups, &c., &c. We have gained much [illegible] formation from this book that we had sought for in vain elsewhere. an economic point of view, $3 cannot be better invested.

---

As spring approaches, those of our lady friends who delight in bea[illegible] fying their homes with flowers and plants will thank us for a suggesti[illegible] founded on experience. In former years we were accustomed to gath[illegible] seeds from our own plants, for use the next year. The consequence [illegible] that our plants were no better than those of our neighbors, who did [illegible] same thing. But during the past few years we have procured fresh [illegible] carefully selected seeds of James Vick, of Rochester, N. Y., and [illegible] success in raising plants from these seeds has convinced us that it is [illegible] only plan which insures success. We shall continue it, and advise friends to do likewise.

